# Grandmother elephants

**DOI:** 10.7554/eLife.01140

**Published:** 2013-07-23

**Authors:** Eve Marder

**Affiliations:** Department of Biology and the Volen National Center for Complex Systems, Brandeis University, Waltham, United Statesmarder@brandeis.edu

**Keywords:** Living science, history of science, neuroscience

## Abstract

As new technology makes it possible to perform experiments that were unimaginable a decade ago, **Eve Marder** argues that we can still learn from the past.

I have never personally met any elephants, although I have seen them walking down the street in Cambodia and India. But my bedspread is full of colourfully stitched elephants and I confess to having acquired a couple of small toy elephants that live on the table next to my bed. It is not that I think of my elephants in a conscious way on a daily basis, but they have a mythic reality for me that is probably best left unchallenged by the reality of meeting one in person.

Why elephants? Many years ago I read that it is old lady elephants, the grandmothers, who maintain the cultural knowledge of their tribes. It is the grandmothers who know where the water holes are; it is the grandmothers who show the younger generations of elephants how to forage for food; and it is the grandmothers who teach their descendants to bury and mourn for their dead. Somehow these stories of cultural knowledge being maintained by the grandmother elephants seemed to dignify the importance of cultural history more palpably than equivalent tales told about and by humans. I don’t really know if the above is myth or reality, or a bit of both, but regardless, it remains a potent metaphor in my mental landscape, and I often find myself musing about the role of our past in the way we conduct science today.

**Figure 1:**
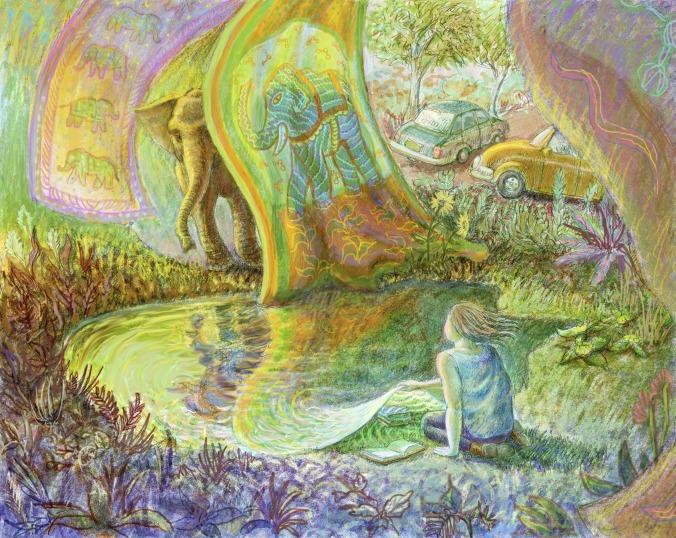
Just as grandmother elephants maintain the cultural knowledge of their tribes, senior scientists should pass on lessons from the history of their field to younger colleagues.

ILLUSTRATION: BEN MARDER

We now face the challenges of a vast onslaught of new papers published every day, sitting in the ether, begging for attention. A diligent student could spend all of his or her time reading just-published papers, grazing in the verdant grasses of new knowledge. And yet, every now and again, I feel the responsibility to be a grandmother elephant, and to remind us all of how we came to know what we know. In particular, I feel the responsibility to remind us all about the impediments to progress back then, so that we have a better idea about the impediments to progress today.

My field, neuroscience, is entering a phase in which remarkable new methods such as optogenetics are allowing researchers to perform experiments that were unimaginable only ten years ago. As part of the NIH Working Group for the BRAIN initiative, I have been stunned and enthralled by the imagination of some of our best scientists as they dream up fantastic new and innovative ways to study brain circuits. The time for the future is now, and it is hard to be anything but enthusiastic about the potential for new discoveries about the brain in health and disease.

At the same time, I feel like a grandmother elephant trying to warn the young that some of the water holes they are moving towards may be mirages or surrounded by quicksand. Part of me thinks that many of those rushing to use new technologies in an effort to understand various circuits within the brain would benefit from knowing more about the hard-fought insights contained in the older literature, and also more about some of the naïve mistakes that were made in the past. I started in neuroscience at a time when ‘circuit cracking’ was done laboriously by poking around with electrodes and hunting for neurons that seemed to be active during specific behaviour. And we learned that circuits are highly interconnected, full of multiple potential pathways by which information can travel, and drastically reconfigured by neuromodulation. These attributes, equally true of the *C. elegans* and human nervous systems, can confound the simple interpretation of genetic and optogenetic manipulations.

However, it seems to me that the path forward is destined to be only modestly influenced by knowing more about the past, as the new methods for circuit analysis are so seductive, powerful and compelling that cautions from history may be functionally irrelevant. Indeed, it may be that warning young researchers about things that happened in the past is never a productive (or even appropriate) response to the enthusiasm and optimism of the present. After all, it is always better to do the experiment and discover the unexpected than to talk yourself out of doing it at all, and discover nothing!

As our technologies become more complex, we sometimes forget to think.

Nonetheless, there is one lesson we can and should take from the past. The early pharmacologists, physiologists and biophysicists were able to extract deep insights into biological processes with simple experiments and clear thinking. A century ago, the British physiologist Thomas Graham Brown anticipated much of what we understand about the spinal cord circuits that are important for walking, and around the same time, many of the fundamental principles of pharmacology were formulated by another British scientist, Henry Hallett Dale, and his colleagues using only bioassays involving muscle movements. As our technologies become more complex, we sometimes forget to think, or our thinking can become fuzzy. Of course, there has always been fuzzy thinking, but just as great music and art survive across generations, while not-so-great music and art fade from our cultural consciousness, I suppose we remember the great scientists of the past and are unburdened by knowledge or memory of their less insightful colleagues.

Maybe the young at heart should just trust their instincts and go willy-nilly into the future.

Modern society is placing enormous pressures on the natural environments in which elephants were wont to roam, and they are changing their behaviour accordingly. (There was a humorous and yet very sad story about a grandmother elephant blocking traffic on a road in India to collect food from motorists trying to pass; the elephant was hungry because humans had long been infringing on her environment, leaving her with few options but to infringe on their environment). One wonders how the cultural roles of those grandmother elephants are altered as their territories are taken. Likewise, I wonder if the fast pace of scientific research today means that the value of lessons from the past has been diminished? Or whether the most important lesson of the past, the value of thinking, is even more important today, as we face almost impenetrable jungles of potential experiments, as we search the unknown for the still-buried treasures of knowledge? There are so many experiments one can do, surely reading and thinking about what is known and how it was learned might help us navigate through the immense possibilities opening before us? Or maybe the young at heart should just trust their instincts and go willy-nilly into the future, unhindered by the detritus of the past?

